# Biochemical Characterization of a Recombinant UDP-glucosyltransferase from Rice and Enzymatic Production of Deoxynivalenol-3-*O*-β-d-glucoside

**DOI:** 10.3390/toxins7072685

**Published:** 2015-07-21

**Authors:** Herbert Michlmayr, Alexandra Malachová, Elisabeth Varga, Jana Kleinová, Marc Lemmens, Sean Newmister, Ivan Rayment, Franz Berthiller, Gerhard Adam

**Affiliations:** 1 Department of Applied Genetics and Cell Biology, University of Natural Resources and Life Sciences, Vienna (BOKU), Konrad Lorenz Str. 24, 3430 Tulln, Austria; E-Mail: gerhard.adam@boku.ac.at; 2 Christian Doppler Laboratory for Mycotoxin Metabolism and Center for Analytical Chemistry, Department of Agrobiotechnology (IFA-Tulln), BOKU, Konrad Lorenz Str. 20, 3430 Tulln, Austria; E-Mails: alexandra.malachova@boku.ac.at (A.M.); elisabeth.varga@boku.ac.at (E.V.); kleinovaja@seznam.cz (J.K.); franz.berthiller@boku.ac.at (F.B.); 3 Department of Chemistry and Biochemistry, Mendel University in Brno, Zemědělská 1, 61300 Brno, Czech Republic; 4 Biotechnology in Plant Production, Department IFA-Tulln, BOKU, Konrad Lorenz Str. 20, 3430 Tulln, Austria; E-Mail: marc.lemmens@boku.ac.at; 5 Department of Biochemistry, University of Wisconsin, 433 Babcock Dr., Madison, WI 53706, USA; E-Mails: snewmist@umich.edu (S.N.); ivan_rayment@biochem.wisc.edu (I.R.)

**Keywords:** masked mycotoxin, glycosylation, sucrose synthase, UDP-glucose recycling, *Fusarium*

## Abstract

Glycosylation is an important plant defense mechanism and conjugates of *Fusarium* mycotoxins often co-occur with their parent compounds in cereal-based food and feed. In case of deoxynivalenol (DON), deoxynivalenol-3-*O*-β-d-glucoside (D3G) is the most important masked mycotoxin. The toxicological significance of D3G is not yet fully understood so that it is crucial to obtain this compound in pure and sufficient quantities for toxicological risk assessment and for use as an analytical standard. The aim of this study was the biochemical characterization of a DON-inactivating UDP-glucosyltransferase from rice (OsUGT79) and to investigate its suitability for preparative D3G synthesis. Apparent Michaelis constants (*K*_m_) of recombinant OsUGT79 were 0.23 mM DON and 2.2 mM UDP-glucose. Substrate inhibition occurred at DON concentrations above 2 mM (*K*_i_ = 24 mM DON), and UDP strongly inhibited the enzyme. Cu^2+^ and Zn^2+^ (1 mM) inhibited the enzyme completely. Sucrose synthase AtSUS1 was employed to regenerate UDP-glucose during the glucosylation reaction. With this approach, optimal conversion rates can be obtained at limited concentrations of the costly co-factor UDP-glucose. D3G can now be synthesized in sufficient quantity and purity. Similar strategies may be of interest to produce β-glucosides of other toxins.

## 1. Introduction

Deoxynivalenol (DON) is the main trichothecene toxin produced by the *Fusarium* species and a relevant virulence factor in Fusarium head blight disease (FHB) of cereal crops. Trichothecene toxins inhibit eukaryotic protein synthesis and elicit a wide range of pathophysiological effects in humans and animals. Examples include immuno-suppression, apoptotic cell death, and aberrant activation of proinflammatory gene expression [1,2]. The effects of DON on the gastrointestinal system, the immune system, and the brain have recently been reviewed [3]. For consumer protection, maximum tolerated levels of DON in grain and food commodities have been enacted in the European Union [4,5], which should prevent a toxin intake higher than the provisional maximum tolerable daily intake (PMTDI) of 1 μg/kg bodyweight for DON and its acetylated derivatives [6]. It was recently shown that at doses close to the PMTDI, DON already exerts a significant influence on intestinal physiology [7,8]. Furthermore, the estimated intake may exceed the PMTDI in years with high *Fusarium* incidence [9] and due to individual dietary preferences [10]. By monitoring the excretion of DON and DON-derivatives in urine, a recent study showed that even when consuming a regular diet, one-third of the participants exceeded the PMTDI of 1 μg/kg bodyweight in Austria [11].

The total exposure to DON (based on DON measured in food commodities) is most likely underestimated due to the occurrence of derivatives originating from plant detoxification systems. Historically, such compounds have been termed “masked mycotoxins”, which implies that they are not routinely detected in standard analytical procedures and may be reactivated to the parental toxins during food processing or digestion [12]. This term has been re-defined recently to be used for plant metabolites of mycotoxins solely [13]. Of particular importance are phase II detoxification metabolites of plants. This route involves conjugation to glucose, malonic acid, or glutathione to form hydrophilic molecules which are stored in vacuoles or transported to the apoplast [14,15]. The actual toxicity of such conjugates for humans and animals is mainly unknown and there is a potential risk that the parental toxins are released through hydrolysis during food processing and in the digestive tract [12,16].

Glycosylation is the major route of phase II detoxification and plants possess a respectable arsenal of UDP-glycosyltransferases (UGT). For example, about 100–180 putative glycosyltransferase family 1 (GT1, [17]) genes have been identified in the genomes of the model plants *Arabidopsis* *thaliana* and *Brachypodium distachyon* [18,19]. Glucosylation effectively reduces the acute toxicity of DON, as demonstrated *in vitro* by reduced inhibition of wheat ribosomes by DON-3-*O*-β-d-glucoside (D3G) [20]. The importance of glycosylation in response to *Fusarium* infections was also indicated by induction of UGT genes in wheat [21,22,23,24]. Increased DON resistance and formation of D3G upon infection with DON has been observed in wheat lines harboring the quantitative trait locus gene Fhb1 [25,26]. However, whether glucosylation of DON is directly responsible for the *Fusarium* resistance of wheat as conferred by Fhb1 has been disputed [27]. Recent evidence clearly shows that overexpression of UGT genes with the ability to detoxify DON leads to increased *Fusarium* resistance in *Brachypodium* [28] and in wheat overexpressing the barley glucosyltransferase HvUGT13248 [29].

D3G is currently present in a wide range of cereal commodities with concentrations typically in the range of up to 20% relative to DON, but higher levels have been reported as well [12,30,31,32]. Efforts to increase *Fusarium* resistance by breeding or through biotechnological approaches may increase the molar ratio of D3G/DON. Yet, little information still exists on bioavailability, metabolism, and long-term toxicity of D3G. It was shown that the bioavailability of D3G is probably low compared to that of DON, as human Caco-2 cells do not absorb D3G [33]. Although D3G is highly resistant to acidic hydrolysis [34], it can be enzymatically hydrolyzed by intestinal bacteria [35] and the released DON may be (partially) absorbed in the distal part of the gut. It was shown that D3G is indeed effectively hydrolyzed in the digestive tracts of humans and animals [36,37,38,39]. Evidence from a pig feeding study indicated that about half of the orally administered D3G is taken up into the bloodstream as DON and is further metabolized [38]. However, a large portion of DON generated by D3G hydrolysis is not resorbed but removed via feces. The European Food Safety Authority (EFSA) Panel on Contaminants in the Food Chain (CONTAM) concluded that with the currently available information, it should pragmatically be assumed that masked or, generally, all modified forms of *Fusarium* toxins possess the same toxicity as the parent compounds [40]. Evidently, D3G is a dietary risk factor and accurate estimation of its toxicological significance is necessary. Toxicological risk assessment can be achieved through feeding trials, but such strategies depend on large amounts of D3G that are currently unavailable.

The aim of this study was to investigate an enzymatic strategy to produce D3G. Several plant UGTs capable of glucosylating DON have been identified in our group previously [18,20,41]. Of these candidates, a rice UGT variant (OsUGT79) could be successfully expressed as an active protein in high yield in *Escherichia coli*. This paper reports the kinetic properties of the recombinant enzyme and its application in the production of D3G. A strategy to limit production costs by UDP-glucose recycling during conversion [42,43] was also successfully employed.

## 2. Results and Discussion

### 2.1. Expression and Purification of OsUGT79

Expression of functional OsUGT79 in *E. coli* was attempted with two fusion-protein variants. The first employed a standard expression system (pET21a) with a *C*-terminal His_6_-tag (UGT-cHis_6_) encoding a protein with a calculated molecular mass of 52 kDa. The second variant was comprised of an *N*-terminal His_6_-tag and a maltose binding protein (MalE) linked to the *C*-terminal OsUGT79 via the Tobacco Etch Virus (TEV) protease recognition site (vector pKLD116, [44]). This protein (nHis_6_-MalE-UGT) has a predicted molecular mass of 95 kDa.

**Figure 1:**
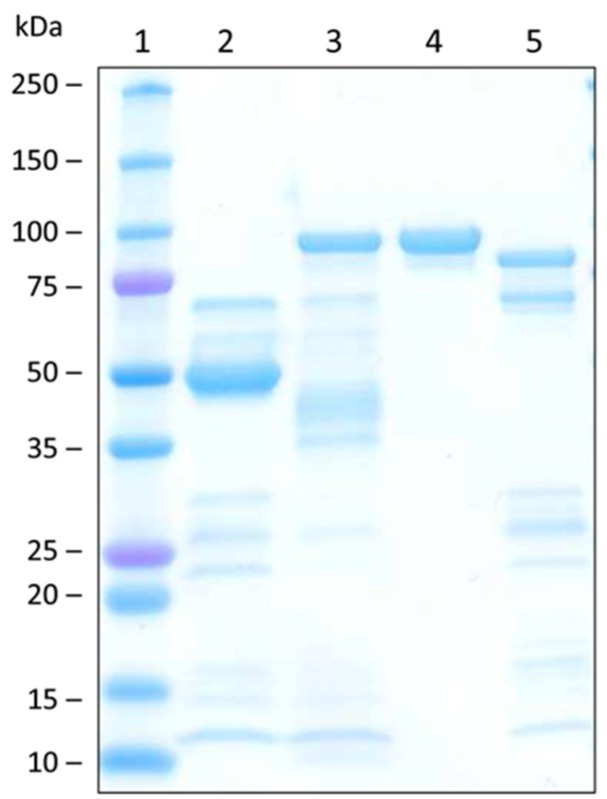
Sodium dodecyl sulfate polyacrylamide gel electrophoresis of the purified rice UDP-glucosyltransferase OsUGT79 and sucrose synthase AtSUS1. Lane 1: Precision Plus Protein Standard (Bio-Rad); lane 2: immobilized metal ion affinity chromatography (IMAC)-purified OsUGT79 (UGT-cHis_6_, 52 kDa); lane 3: IMAC-purified OsUGT79 (nHis_6_-MalE-UGT, 95 kDa); lane 4: nHis_6_-MalE-UGT after size exclusion chromatography; lane 5: IMAC-purified AtSUS1 (93 kDa).

Protein expression levels were estimated from the total protein (Bradford assay) obtained after one-step purification by immobilized metal ion affinity chromatography (IMAC). First experiments with *E. coli* BL21 yielded low expression levels of <7 mg protein per liter broth with both constructs. Expression could be improved with *E. coli* Rosetta: 30 mg of protein per liter (4.4 mg per g of wet biomass) were obtained with nHis_6_-MalE-UGT and 85 mg per liter (5.7 mg per g of wet biomass) with UGT-cHis_6_. This implies an almost three-fold better yield of the construct without the maltose binding protein. However, activity measurements (1 mM DON/10 mM UDP-glucose) suggested that with a specific activity of 0.15 μmol min^−1^ mg^−1^ (referring to D3G formation), IMAC-purified nHis_6_-MalE-UGT is much more active than UGT-cHis_6_ with only 0.015 μmol min^−1^ mg^−1^. Despite the lower expression levels, the solubility enhancing maltose binding protein MalE appears to improve the yield of active protein. Therefore, further experiments were performed only with nHis_6_-MalE-UGT. The IMAC fraction of this protein was further purified by size exclusion chromatography (SEC). The specific activity (1 mM DON/10 mM UDP-glucose) of the purest fraction (Figure 1, lane 4) was 0.28 μmol min^−1^ mg^−1^.

### 2.2. Kinetic Characteristics of OsUGT79

**Figure 2:**
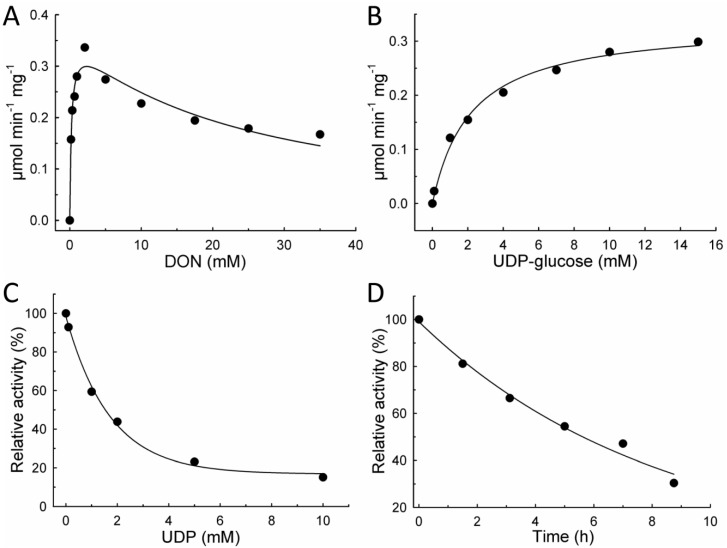
Kinetic characterization of recombinant OsUGT79 (nHis_6_-MalE-UGT) at 37 °C, 100 mM Tris pH 7. (**A**) 10 mM UDP-glucose, varying deoxynivalenol (DON) concentrations; (**B**) 1 mM DON, varying UDP-glucose concentrations; (**C**) Inhibition by UDP (1 mM DON); (**D**) Stability at 37 °C in 100 mM Tris pH 7.

Kinetic characterization of OsUGT79 was performed with SEC-purified enzyme. Increasing the concentration of DON at a constant UDP-glucose concentration of 10 mM revealed substrate [S] inhibition by DON (Figure 2A). Maximum reaction velocity (0.34 μmol min^−1^ mg^−1^) was observed at 2 mM DON. Regression analysis using the kinetic model of Haldane (Equation (1)) yielded an apparent Michaelis constant (*K*_m_) of 0.23 ± 0.06 mM DON and a theoretical *V*_max_ of 0.36 ± 0.02 μmol min^−1^ mg^−1^, corresponding to a *k*_cat_ of 0.57 s^−1^. Substrate inhibition by DON was characterized by an estimated inhibitory constant (*K*_i_) of 24 ± 5 mM. Response to variable UDP-glucose concentrations (1 mM DON) can be described with the typical Michaelis-Menten model (Equation (2), Figure 2B) with a *K*_m_ of 2.2 ± 0.3 mM UDP-glucose. Inclusion of UDP in enzyme assays (1 mM DON/10 mM UDP-glucose) indicated that UDP is an effective inhibitor of OsUGT79 (Figure 2C). Using the model of exponential decay (Equation (3); *v*_(I)_ reaction velocity as function of inhibitor concentration [I], *v*_min_ velocity at maximum inhibitor concentration ([I]→∞), *v*_0_ velocity at [I] = 0, λ decay constant), 50% activity reduction was estimated at 1.5 mM UDP. The stability of the enzyme was tested by incubation at reaction conditions (100 mM Tris, 37 °C). Samples were taken in regular intervals and assayed for activity with DON (Figure 2D). This revealed a half-life of 5–6 h at 37 °C (Equation (4); *v*_(*t*)_ reaction velocity at incubation time *t*, *v*_0_ initial velocity at *t* = 0).

(1) $$v=\;\frac{V_{\text{max }}\;\left[S\right]}{K_{\text{m}}\;+\left[S\right]+\frac{S^{2}}{K_{\text{i}}\;}}$$

(2) $$v=\;\frac{V_{\text{max }}\;\left[S\right]}{K_{\text{m}}\;+\left[S\right]}$$

(3) $$v_{\left(\text{I}\right)}=\;v_{\text{min}}+\;v_{0}\;e^{-\text{λ}\left[\text{I}\right]}$$

(4) $$v_{\left(t\right)}=\;v_{0}\;e^{-\text{λ}t}$$

Divalent cations can have an influence on the activity of glycosyltransferases [45]. Structural studies of a GT-B fold UGT showed that Ca^2+^, Mn^2+^, and Mg^2+^ can interact with the β-phosphate group of UDP and thus possibly play a role in facilitating product release [46,47]. Addition of ethylenediaminetetraacetic acid (EDTA) in the assay caused only low reduction of activity (Table 1), implying that OsUGT79 does not strongly depend on metal ions. Ca^2+^, Mg^2+^, and Fe^2+^ caused a moderate increase of activity (Table 1). Strong inhibition of a UGT by Cu^2+^, Mn^2+^, and Zn^2+^ (1 mM) was previously reported [48]. Here, complete inhibition of OsUGT79 was observed by Cu^2+^ and Zn^2+^, but not by Mn^2+^ (all 1 mM), which increased activity to about 140%.

**Table 1:** Influence of ethylenediaminetetraacetic acid (EDTA) and several metal ions (1 mM each) on the activity of OsUGT79. ND, deoxynivalenol-3-*O*-β-d-glucoside not detectable.

Compound	Activity (%)
Control	100
EDTA (1 mM)	98
EDTA (5 mM)	95
CaCl_2_	108
CuSO_4_	ND
MgSO_4_	117
MnCl_2_	143
MnSO_4_	139
ZnSO_4_	ND
FeSO_4_	114

### 2.3. UDP-Glucose Recycling and DON Production

A prerequisite for the preparative production of D3G is a complete conversion, with residual DON concentrations below 1%. In order to achieve this within a reasonable time frame, a considerable excess of UDP-glucose would be required to maintain reaction velocity, especially to compensate for feedback inhibition by UDP accumulating during the reaction.

**Figure 3:**
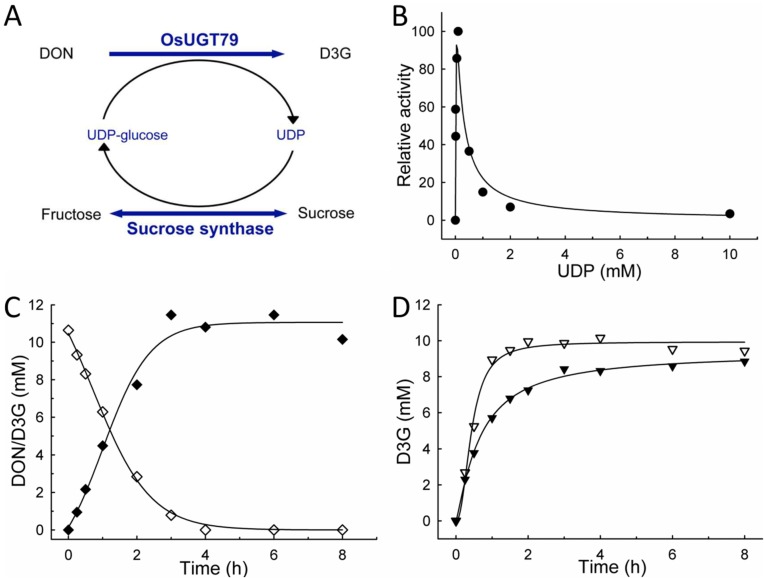
*In situ* UDP-glucose recycling with OsUGT79 and sucrose synthase AtSUS1 from *Arabidopsis thaliana*. (**A**) Reaction scheme; (**B**) Initial reaction velocities (1 mM deoxynivalenol (DON), 100 mM sucrose) resulting from different UDP concentrations, 0.2 mg mL^−1^ of each protein in assay; (**C**) Glucosylation of 10 mM DON with 0.1 mM UDP, 1 mg mL^−1^ of each protein in assay. All assays performed at 37 °C, 100 mM Tris pH 7, DON (◊), DON-3-*O*-β-d-glucoside (D3G; ♦); (**D**) Glucosylation of 10 mM DON with 10 mM UDP-Glucose at 37 °C, 100 mM Tris pH 7, comparison of reaction with (▽) and without (▼) recycling of UDP-glucose, OsUGT79/AtSUS1 each 1 mg mL^−1^ in assay.

Based on previous studies [42,43], the potential of a sucrose synthase from *Arabidopsis* *thaliana* (AtSUS1) was investigated in order to recycle UDP-glucose during DON glucosylation, and the reaction scheme is illustrated in Figure 3A. AtSUS1 catalyzes the reversible formation of UDP-glucose from sucrose and UDP with *K*_m_ values of 53 and 1.2 mM, respectively [43]. In the reversed sucrose synthesizing reaction, *K*_m_ values of 25 mM fructose and 50 μM UDP-glucose were reported [49].

In order to establish whether this strategy is applicable for D3G production, initial experiments were performed in the absence of UDP-glucose, but with 100 mM sucrose and different initial UDP concentrations. Both proteins (OsUGT79 and AtSUS) were applied at concentrations of 0.2 mg mL^−1^ (IMAC purification stage, Figure 1). The results (Figure 3B) show that D3G formation rates strongly depend on initial UDP concentrations. In agreement with the strong inhibition of OsUGT79 by UDP, maximum conversion rates were found at low UDP concentrations of 0.1 mM. Regression analysis (Equation (1)) implied that the reaction can be described analogous to an enzyme subject to substrate inhibition. An apparent *K*_m_ of 0.014 mM and a *K*_i_ of 0.18 mM UDP were estimated. Therefore, maximum conversion rates occurred at virtual UDP-glucose concentrations (<0.1 mM) that are at least 20-fold below the *K*_m_ (2 mM). This would imply that using UDP as sole substrate is of low practical value due to kinetic limitations. Nevertheless, with 0.1 mM UDP, 10 mM DON were rapidly converted to D3G (Figure 3C), with residual DON (molar ratio DON/D3G) below 1% after 6 h.

In the case that an aglycon is not stable in aqueous conditions, it is essential to complete the reaction as fast as possible. Rapid conversion is also of interest in view of the limited half-life of OsUGT79 (5–6 h, Section 2.1) at reaction conditions. Therefore, recycling of UDP-glucose is also of interest to maintain high donor concentrations and to avoid accumulation of UDP during the reaction. To demonstrate this, glucosylation of 10 mM DON with equimolar initial UDP-glucose concentration was compared with and without UDP-glucose recycling. The results (Figure 3D) confirm that inclusion of AtSUS1 is effective to maintain optimal reaction conditions. With the aid of donor recycling, glucosylation was almost completed within 2 h. In absence of AtSUS1, the reaction velocity declined rapidly.

### 2.4. Preparative Production and Purification of D3G

A typical batch for larger scale D3G production contained 50–100 mg DON with concentrations of 10 mM. Reaction conditions were as described above with 1 mg mL^−1^ OsUGT79 (IMAC purification stage). UDP-glucose concentrations were 2 mM (concentration at *K*_m_) when AtSUS1 was included. Batches without donor recycling contained UDP-glucose in 1.5 molar excess over DON. The reactions were usually carried out overnight.

**Figure 4:**
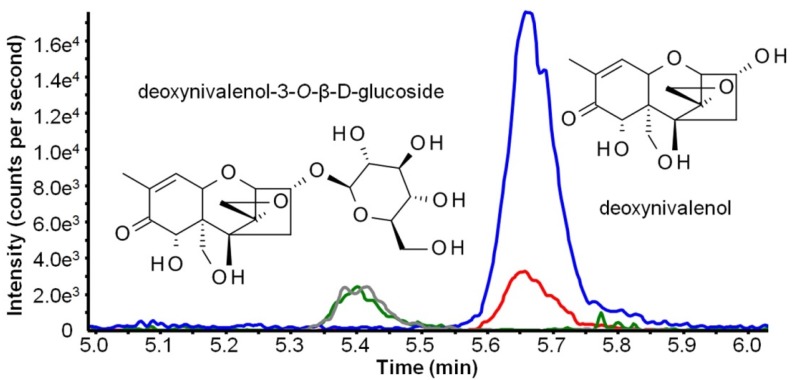
Structure formula and extracted ion chromatogram of a standard containing 100 μg L^−1^ deoxynivalenol (DON) and DON-3-*O*-β-d-glucoside (D3G). Green (*m*/*z* 517.3 > *m*/*z* 59.1) and gray (*m*/*z* 517.3 > *m*/*z* 427.1) lines show selected reaction monitoring (SRM) traces for D3G. Blue (*m*/*z* 355.1 > *m*/*z* 59.2) and red (*m*/*z* 355.1 > *m*/*z* 265.2) lines show SRM traces for DON.

High performance liquid chromatographic-mass spectrometric (HPLC-MS/MS) measurements were performed prior to purification to confirm successful and complete conversion of DON to D3G. Both selected reaction monitoring (SRM) transitions of D3G were present at a retention time of 4.97 min and DON was not found in the final product (<0.05%). A chromatogram of a standard containing 100 μg/L of both compounds is shown in Figure 4.

Purification was carried out on a preparative high performance liquid chromatography system and the obtained yield of D3G in a crystalline form after evaporation and lyophilization was 53 mg for a batch containing 50 mg DON. A subsample of the crystalline D3G was dissolved in MeOH and its purity was determined as follows: The overall purity was determined by HPLC-ultraviolet (UV) determination at 200 nm and was above 98% based on a 500 mg L^−1^ solution, which is in agreement with the used DON. The absence of DON was confirmed by LC-MS measurements and verified to be below the limit of detection (corresponding to <0.05% overall). Furthermore, enhanced product ion (MS/MS) scans were performed at three collision energies (10, 20, 30 eV). Comparison with MS/MS measurements of a commercially available certified D3G standard under the same conditions proved the compounds to be identical.

## 3. Experimental Section

### 3.1. Materials

Uridine 5′-diphosphoglucose disodium salt hydrate (UDP-glucose) from *Saccharomyces cerevisiae* (cat. no. U4625) and Uridine 5′-diphosphate disodium salt hydrate (UDP, cat. no. 94330) were purchased from Sigma-Aldrich (Vienna, Austria). *E. coli* BL21 Star™ (DE3) was from Invitrogen (Carlsbad, CA, USA), *E. coli* Rosetta™ (DE3) from Novagen (Madison, WI, USA). DON (purity >98%) was purified at the IFA-Tulln following a published procedure [50].

### 3.2. Cloning of OsUGT79 and AtSUS1

The OsUGT79 gene (GenBank accession NM_001058779) was amplified from plasmid pWS57 [18] using the forward primer 5′-ATGGGCTCTATGTCCACTCCTGC-3′ and the reverse primer 5′-ATTGGAATACTTTGCTGCAAACTC-3′. Using the Quikchange method [51,52], the resulting product was introduced into pET21a (Novagen, Madison, WI, USA) and plasmid pKLD116, a pET31b derivative containing His_6_-tagged maltose binding protein (MalE), followed by a TEV protease cleavage site [44].

The sucrose synthase gene (AtSUS1) from *Arabidopsis thaliana* (TAIR accession AT5G20830.1; GenBank accession BAH19538.1) was amplified from *A. thaliana* cDNA using the forward primer 5′-CATATGGCAAACGCTGAACGTATG-3′ and the reverse primer 5′-GCGGCCGCGTCATCTTGTGCAAGAGG-3′. The restriction sites *Nde*I and *Not*I were used for cloning into pET21a in frame with the *C*-terminal His-tag. AtSUS1 was expressed with *E. coli* BL21 Star.

### 3.3. Protein Expression and Purification

Protein expression was carried out in terrific broth with 100 mg L^−1^ ampicillin (*E. coli* BL21 (DE3)), additionally supplemented with 35 mg L^−1^ chloramphenicol for *E. coli* Rosetta. Isopropyl-β-D-1-thiogalactopyranoside (IPTG, 0.5 mM final concentration) was added when the optical density (OD_600_) reached 0.5. The flasks were further incubated for 16 h at 25 °C and 100 rpm. After that period, the biomass was harvested by centrifugation (4000 *g*, 15 min) and resuspended in 25 mM Tris pH 7.5 + 500 mM NaCl/25 mM imidazole, the binding buffer for the first purification step. The cells were disrupted in a French Pressure Cell Press (Aminco, Silver Spring, MD, USA), in three passes at 1200 psi (*ca.* 8270 kPa). The cell extract was cleared by centrifugation at 70,000 *g*. Protein purification was performed by IMAC on Ni^2+^-charged HisTrap Crude FF columns, 5 mL (GE Healthcare, Vienna, Austria). Target protein was bound to the column in the above specified binding buffer. Protein was eluted with the same buffer containing 500 mM imidazole. After IMAC, the buffer was changed to 50 mM potassium phosphate pH 7 + 150 mM NaCl by gel filtration with Sephadex G25 (GE Healthcare). OsUGT79 was stored as such or further purified by size exclusion chromatography on Superose 12 (GE Healthcare) with 50 mM potassium phosphate pH 7 + 150 mM NaCl. Column dimensions were 2 cm^2^ area, 90 cm bed height, flow 0.5 cm min^−1^. Purified OsUGT79 (IMAC purification stage) was stored at −80 °C. AtSUS1 almost completely lost activity by freezing/thawing and had to be prepared fresh prior to use.

Protein concentrations were determined with the Bio-Rad (Vienna, Austria) protein assay based on the dye-binding method of Bradford. Sodium dodecyl sulfate polyacrylamide gel electrophoresis (SDS-PAGE) including Coomassie blue staining was performed with the Mini-PROTEAN system with precast gels (4%–20%) from Bio-Rad. The molecular mass marker used was High Precision Dual Color (10–250 kDa range, Bio-Rad).

### 3.4. Glycosylation Assays

Unless mentioned otherwise, enzyme assays were performed in 100 mM Tris, pH 7 at 37 °C, 10 min reaction time, OsUGT79 was added to concentrations of 0.1–1 mg mL^−1^. Assays for UDP-glucose recycling with AtSUS1 additionally contained 100 mM sucrose. The assays were stopped by transferring 20 μL of sample to 180 μL methanol. After centrifugation (20,000 *g*, 5 min) to remove precipitated protein, the samples were further diluted in H_2_O to an expected concentration range of 1 mg L^−1^ DON/D3G. The concentrations of DON and D3G were determined by HPLC-MS/MS (see Section 3.5). The activity units (μmol min^−1^ mg^−1^) displayed refer to the formation of D3G per mg of protein. All assays were performed in duplicate. Data analysis (*i.e.*, for kinetic enzyme characterization) was performed with SigmaPlot 11.0 (Systat Software, San Jose, CA, USA) using Equations (1)–(4).

### 3.5. DON and D3G Determination by HPLC-MS/MS

Concentrations of DON and D3G were determined on a 1290 HPLC system coupled to a 4000 QTrap LC-MS/MS System (AB Sciex, Foster City, CA, USA). Briefly, chromatographic separation was achieved on a Gemini C18 column (150 × 4.6 mm, 5 μm, Phenomenex, Aschaffenburg, Germany) at 25 °C with a flow rate of 0.8 mL min^−1^. The following water-methanol gradient (eluent A: 80:20, *v*:*v*; eluent B: 3:97, *v*:*v*; both containing 5 mM ammonium acetate) was used: initial conditions at 0% B were held for 1 min, followed by a linear increase to 50% B within 5 min. Afterwards, 100% B were reached within 0.1 min. Following a holding time of 2 min at 100% B, a fast switch to the initial conditions was performed and column equilibration was achieved until the end of the run (10 min). The mass spectrometer was operated in negative electrospray ionization mode. The following source settings were used: temperature 550 °C, ion spray voltage −4 kV, curtain gas 30 psi (207 kPa of 99.5% nitrogen), source gas one and two both 50 psi (345 kPa of zero grade air), collision gas (nitrogen) set to high. For quantitation two SRM transitions per compound were acquired with a dwell time of 25 ms. The acetate adducts (*m*/*z* 355.1 for DON, *m*/*z* 517.3 for D3G) were chosen as precursors and the declustering potential (DP) was −40 V for DON, −50 V for D3G. The product ions were for DON *m*/*z* 59.2 (collision energy (CE) of −40 V) and *m*/*z* 265.2 (CE −22 V), for D3G *m*/*z* 427.1 (CE −30 V) and *m*/*z* 59.1 (CE −85 V).

### 3.6. Preparative High-Performance Liquid Chromatography

Purification of D3G was carried out using an 1100 series preparative HPLC system equipped with an automatic fraction collector and a multiple wavelength detector (MWD) (all Agilent Technologies, Waldbronn, Germany). A Gemini NX column (150 × 21.2 mm, 5 µm, Phenomenex, Aschaffenburg, Germany) and gradient elution (eluent A: water, eluent B: methanol) was used for the separation of D3G from residual glucose and other impurities. The initial conditions of 20% B were maintained for 1 min, followed by a linear increase to 60% B within 4 min and to 100% B within 0.1 min. Following a hold time of 1 min at 100%, the initial conditions were achieved with a fast switch to 25% B and the column was equilibrated prior to the next injection. The flow rate was 16 mL min^−1^ and the injection volume was set to 900 μL, in general. The fractions were collected from 4 to 6 min with the maximum peak duration of 0.5 min using threshold working mode. The collected fractions were pooled, the organic phase was evaporated on a rotary evaporator at 30 °C, and the rest of water phase was removed by lyophilization. The obtained crystals of D3G were weighed in a glass vial on a micro balance and stored at −20 °C.

### 3.7. D3G Purity Analysis by HPLC-UV

For the purity measurement of the crystalline D3G, an 1100 Series HPLC system (Agilent Technologies, Waldbronn, Germany) including an 1100 Series HPLC MWD and a Gemini C18 column (150 × 4.6 mm, 5 μm, Phenomenex, Aschaffenburg, Germany) was used. Pure water (A) and acetonitrile (B) were used as eluents and the following gradient was applied: 0–1 min 5% B; 1–26 min linear increase to 100% B, 26–29 min 100%, 29.1–32 min 5% B. During the whole run the UV absorption was measured at the wavelengths 200 nm, 210 nm, and 220 nm, but only the 200 nm signal was used for the purity assessment. The final product of D3G was weighed with a microbalance and dissolved in methanol to obtain a concentration of *ca.* 500 mg L^−1^. Of this solution 10 μL were injected into the system and the peak area of D3G was divided by the sum of all peak areas and multiplied with 100 to obtain the overall purity in percent.

## 4. Conclusions

This paper reports the kinetic characteristics of a DON-conjugating UDP-glucosyltransferase from rice (OsUGT79), which can be used for preparative synthesis of D3G. The fact that this enzyme can be functionally expressed with *E. coli* is a great advantage. Previously, engineered yeast strains expressing plant UGTs were employed to produce β-glucosides of zearalenone and its derivatives in the mg range [53,54]. The availability of a functional cell-free catalyst simplifies the production process drastically and allows catalysis to be performed in small volumes and, most importantly, facilitates product clean-up. Recycling of UDP-glucose is an effective strategy to optimize conversion kinetics and is of economic interest to reduce the required amounts of the costly co-factor UDP-glucose. Therefore, it is now possible to provide sufficient amounts of D3G for toxicological risk assessment by a relatively simple and rapid procedure. Similar approaches may be of interest to produce β-glucosides of other mycotoxins that are so far not commercially available.
